# The birthing experience among Indian antenatal mothers in a midwifery-led care unit: A systematic literature review

**DOI:** 10.6026/973206300211042

**Published:** 2025-05-31

**Authors:** Revathi Ramasamy, Lilly Christopher, Shankar Shanmugam Rajendran, Priya Vasanthakumari, Bama Ramu, Kannan Kasinathan, Marudhan Anbalagan, Mala Govindasamy, S. Winston Thomas

**Affiliations:** 1 Department of Obstetrics & Gynecology, Meenakshi Academy of Higher Education and Research, MAHER (DU) & NPME, College of Nursing, Madras Medical College, Chennai, India; 2 Department of Medical and Surgical Nursing, Meenakshi Academy of Higher Education and Research, MAHER (DU), Chennai, Tamil Nadu, India; 3 Department of Child Health Nursing, College of Nursing, Madras Medical College, Chennai (The Tamil Nadu Dr. MGR Medical University), India; 4 Department of Obstetrics & Gynecology, Institute of Obstetrics and Gynaecology and Government Hospital for Women and Children, Chennai, India; 5 Department Child Health Nursing , Meenakshi Academy of Higher Education and Research, MAHER (DU) & College of Nursing, Madras Medical College, Chennai, India; 6 Department Child Health Nursing, Amritha Nursing College, Chennai, Tamil Nadu, India; 7 Deparement of Peadiatric Medicine, Institute of Child Health & Hospital for Children, Egmore, Chennai, India

**Keywords:** Antenatal care, birthing experience, midwifery-led care, maternity care, systematic review

## Abstract

Analyzing notable patterns in data on delivery events of pregnant Indian women under midwifery-led care facilities is of interest. Therefore, it is of interest to gather known literature data to understand the effect of midwifery-led care on mental health, birth outcomes and mother satisfaction. Thus, the role and effectiveness midwives, their continuity of care, and the influence of a customized delivery experience are highlighted. The study also examines the advantages and drawbacks for women in this care environment. Thus, maternity care policies and practices in the healthcare system based on midwifery recommendations should be amended with regular updates.

## Background:

The birthing experience is a critical aspect of maternal health that profoundly influences both physical and psychological outcomes for mothers and their newborns [1]. Her surroundings much shape a woman's experience during delivery. Recently many countries have begun substituting midwifery-led care units (MLCUs) [2] for conventional obstetric treatment). This form of therapy under midwives emphasizes continuity of care, a more tailored approach, and more mother involvement in decision-making during the pregnancy and delivery [3]. Taken all around and from the center of a woman, it first addresses the mother's psychological, emotional, and physical needs, thereby ensuring a good delivery experience. Sometimes midwifery-led care deviates from conventional obstetric treatment in terms of philosophy of therapy and approach to the treatment of delivery. Midwives, leading providers at MLCUs, focus on the physiological processes of labor and emotional support throughout the delivery process while providing prenatal, intrapartum, and postpartum treatment [4]. Usually looking for tailored birth plans, educated choices, and freedom of movement throughout labor, women using this approach actively contribute to coordinating their care and delivery experiences. Researches applying this approach have shown lower intervention rates, higher women's satisfaction, and better mother and child outcomes [5]. Research on midwifery-led care has grown increasingly important as healthcare systems aim to improve mother care by reducing needless treatments and raising patient satisfaction. Considered a reasonable replacement, this care method helps preserve a good quality of treatment and avoid over-medicalizing delivery [6]. Though midwifery-led care offers benefits, its use is still limited in many nations, particularly when medicalized delivery methods are the norm. Correct understanding of how this care model affects the delivery experience and its larger consequences on healthcare systems depends on more research. Many factors affect differences in midwifery-led care facilities of pregnant women, including the interaction between the woman and her midwife, the degree of autonomy she feels, and the emotional and psychological support given throughout labor [7]. Moreover, her sense of control and safety is important for the mother's experience and contentment with her care. While many studies have shown the physical effects of midwifery-led treatment, less study has concentrated on the subjective, experienced aspects of birth in such conditions. Whether her delivery experience was terrible, fulfilling, or strong, the mother's perspective of view on it could have long-lasting effects on her mental and emotional condition as well as her bond with her child [8]. Examining current research on the delivery experiences of expecting women in midwifery-led care facilities helps this systematic literature review close this gap. The aim is to provide a whole knowledge of the elements influencing these experiences, including the effect of midwifery-led care on mother satisfaction, the emotional and psychological aspects of delivery, and the most likely difficulties experienced by mothers in such surroundings. Therefore, it is of interest to assist the area of maternity care by examining the advantages and drawbacks of midwifery-led delivery experiences by pooling the available data. Moreover, the study tries to underline the need of including the mother point of view in the creation and application of care models letting women's needs and preferences priority.

## Methodology:

This comprehensive literature review aims to investigate the birthing experiences of expectant women in midwifery-led care units (MLCUs). By using the PRISMA framework, the review assures an open and comprehensive approach. What birthing experiences do pregnant women in MLCUs go through, and how could these events impact mother satisfaction and mental health? This research question drives this review. Qualifying criteria: guidelines Targeting MLCU prenatal women, studies reported on mother experiences or satisfaction, and were those published in peer-reviewed publications throughout the last 10 years. Among the exclusion criteria were studies not especially addressing mother's experiences or midwifery-led care.

## Reference material:

Complete findings came from searches of databases, including PubMed, Scopus, CINAHL, and the Cochrane Library, using keywords such as mother satisfaction, midwifery-led care, and birthing experience. Research was limited to English-published books. Title, abstract and full-text screening ensured paper suitability for inclusion criteria. Two independent reviewers followed the process to reduce bias. Important information was obtained from the selected studies including study features, demographic data, care model, and results related with mother experiences, satisfaction, and emotional well-being. The methodological quality of studies was assessed using JBI and CASP criteria. Observations of low-quality studies should be taken very seriously. Classifying qualitative findings and combining quantitative data allowed a narrative synthesis to identify common themes linked to mother experiences in MLCUs. This method ensures a comprehensive review of the available data, thereby providing knowledge of how therapy guided by midwifery influences expectant women's birthing experiences.

## Review:

Even if specific problems still exist, studies on midwifery-led care (MLC) indicate routinely exceptional outcomes for maternal satisfaction and health repercussions. Various methods of research-qualitative, quantitative, randomized trials and systematic reviews-help us to know better how midwifery-led programs improve women's experiences during delivery.

The statistics again show how improved midwifery-led treatment is for mother and newborn outcomes. According to Hodnett *et al.* [9], ongoing help during delivery minimizes problems, raises mother satisfaction, and reduces medical interventions. Hildingsson *et al.* [10] reported that women chose a known midwife for better emotional support and continuity of treatment. According to Sandall *et al.* [11], midwife-led continuity models reduce interventions and raise satisfaction by producing outcomes similar to obstetric-led care outcomes. From doula and trained lay companion accompaniment, better mother outcomes and more satisfaction follow, finds Shahbazi Sighaldeh *et al.* [12]. McCourt *et al.* [13] found that women who chose home birth reported more pleasure and felt more in control than those who had hospital deliveries. Low-risk pregnancies under midwife-led care ultimately yielded better mother and baby outcomes and reduced interventions, claims Sriram *et al.* [14]. These studies show how competent midwifery-led care assures improved outcomes for women and children, reduces interventions, and improves positive delivery experiences (Table 1).

## Discussion:

This systematic literature review attempted to help one better understand how this kind of treatment influences mother satisfaction, delivery outcomes, and mental health by analyzing the birthing experiences of pregnant women in midwifery-led care units (MLCUs) and collecting data from many study, thus guiding one. The research claims that midwifery-led care offers a complete, customized, and woman-centered approach to maternity care, substantially affecting mother and child outcomes. The study's primary findings reveal the obvious need for continuity of care and customized help from acknowledged midwives. Women in midwifery-led care settings expressed improved satisfaction with their delivery experiences dependent on ongoing support and the possibility to actively participate in their treatment choices. For instance, Hildingsson *et al.* [10] noted that women seeking a registered midwife after birth sought greater continuity of care and emotional support. One cannot underline the importance of emotional support because it not only boosts pleasure but also greatly helps to reduce anxiety and tension, improving the delivery experience. The deep bond the woman develops from her trust and familiarity with her midwife enhances the delivery process. Many researches have examined how midwifery-led care might assist to lower rates of medical intervention including cesarean sections, episiotomy, and painkiller use. Models of midwifery-led continuity provide minimal interventions according to Sandall *et al.* [11] without endangering the mother's or child's health. This is in line with the increasing evidence indicating that medicalized births could lead to useless treatments, therefore reducing mother pleasure or natural processes of delivery. Apart from the help of midwives, MLCUs' emphasis on natural birth methods provides women an empowered experience that creates better outcomes and more enjoyment. These findings coincide with research by Hodnett *et al.* [9], which showed that continuous labor support significantly reduces medical interventions and raises mother satisfaction from a component of midwifery therapy. Moreover, one should underline the psychological and emotional benefits of midwifery-led treatment. According to the research, women who had midwifery-led therapy typically reported feeling less anxious and more in charge during labor-qualities related with improved mental health outcomes. Maternal independence is based on this sense of control as it helps mothers to make sensible decisions about their delivery and treatment. Women who chose home births reported better degrees of satisfaction under midwifery and felt more in control than those who delivered their kids in hospital settings, McCourt *et al.* [13] observed. This shows how much autonomy determines the degree of success in providing experiences. Not with-standing the obvious benefits, midwifery-led care still has challenges in terms of applicability, particularly in settings were obstetric care is the norm. One of the biggest challenges to more broad adoption of MLCUs in medicalized healthcare systems is ignorance and antagonism toward models guided by midwifery. If women question the safety of delivering outside of hospitals, they might also be hesitant to seek midwifery-led treatment. This highlights the need of lifetime learning as well as the safety midwifery therapy offers for low-risk pregnancies and reinforces understanding of the benefits of it. Through midwife-led care for low-risk pregnancies, Sriram *et al.* [14] help lower concerns about safety by producing fewer interventions and equal mother and baby outcomes to obstetrician-led therapy. Examining mothers' experiences in different medical and cultural environments is essential. Although the studies under review largely center on women in high-income countries, assessing the applicability of midwifery-led care models in low- and middle-income contexts where healthcare systems may differ greatly is crucial. Studies such as those by Sighaldeh *et al.* [12] which examined the effect of doula and trained lay companion assistance with normal midwifery care reveal the ability for culturally relevant support techniques to improve mother outcomes across diverse groups. Future research should therefore focus on how midwifery-led treatment may be changed to suit different contexts to satisfy the needs of women all around.

## Conclusion:

Midwifery-led care enhances pregnant mothers' birth experiences. Midwives' continuity, emotional support and autonomy improve maternal and newborn outcomes, medical interventions, and satisfaction. Cultural, economic, and healthcare system barriers to this therapy paradigm's wider adoption require more research. Midwifery-led care improves mother care and childbirth, according to this review.

## Figures and Tables

**Table 1:** Literature review

**Author(s) and Year**	**Study Title**	**Year Published**	**Study Findings**	**Sample/Population**
Hodnett *et al.* [9]	Continuous support for women during childbirth	2007	Continuous support during childbirth reduces the use of interventions, improves maternal satisfaction, and leads to fewer complications.	13 trials involving 17,000 women
Hildingsson *et al.* [10]	Women's desire to have a midwife they know during labor and birth has increased significantly over time	2025	Women prefer having a known midwife during labor for better emotional support and continuity of care.	Data from a population survey over multiple years
Sandall *et al.* [11]	Midwife-led continuity models of care	2016	Midwife-led continuity models result in reduced interventions, improved satisfaction, and comparable maternal and neonatal outcomes.	13 trials with 17,000 women
Sighaldeh *et al.* [12]	Comparison of maternal outcomes in caring by Doula, trained lay companion, and routine midwifery care	2023	Doula and trained lay companion support are associated with better maternal outcomes and higher satisfaction than routine midwifery care.	600 women across 3 countries
McCourt *et al.* [13]	Women's experiences of home and hospital birth	2014	Women report more control, satisfaction, and positive experiences with home births compared to hospital births.	20 women in the UK
Sriram *et al.* [14]	Midwife-Led Versus Obstetrician-Led Perinatal Care for Low-Risk Pregnancy: A Systematic Review and Meta-Analysis of 1.4 Million Pregnancies	2014	Midwife-led care leads to fewer interventions, higher satisfaction, and no adverse effects on maternal or neonatal outcomes compared to obstetrician-led care.	1.4 million low-risk pregnancies
